# Supplementary material for: Pyruvate Dehydrogenase Kinase 1 inhibition mediated oxidative phosphorylation enhancement in cartilage promotes osteoarthritis progression
Source: BMC Musculoskelet Disord. 2023 Jul 20;24:597. doi: 10.1186/s12891-023-06585-6 (PMC10357736; doi:10.1186/s12891-023-06585-6)
Supplement: Supplementary file 1 — Additional file 1. [file 12891_2023_6585_MOESM1_ESM.zip › KEGG.Gsea.1653622432464/KEGG_LEUKOCYTE_TRANSENDOTHELIAL_MIGRATION.html]

Details for gene set KEGG\_LEUKOCYTE\_TRANSENDOTHELIAL\_MIGRATION[GSEA]

|  || Dataset | 55457\_collapsed\_to\_symbols.cls.cls #OA\_versus\_normal.cls.cls #OA\_versus\_normal\_repos |
| Phenotype | cls.cls#OA\_versus\_normal\_repos |
| Upregulated in class | normal |
| GeneSet | KEGG\_LEUKOCYTE\_TRANSENDOTHELIAL\_MIGRATION |
| Enrichment Score (ES) | -0.28990158 |
| Normalized Enrichment Score (NES) | -1.2779751 |
| Nominal p-value | 0.0912 |
| FDR q-value | 0.30311707 |
| FWER p-Value | 1.0 |
Table: GSEA Results Summary

  

Fig 1: Enrichment plot: KEGG\_LEUKOCYTE\_TRANSENDOTHELIAL\_MIGRATION      
 Profile of the Running ES Score & Positions of GeneSet Members on the Rank Ordered List

  

| SYMBOL | TITLE | RANK IN GENE LIST | RANK METRIC SCORE | RUNNING ES | CORE ENRICHMENT || 1 | CYBA | cytochrome b-245 alpha chain [Source:HGNC Symbol;Acc:HGNC:2577] | 501 | 0.658 | -0.0180 | No |
| 2 | VAV1 | vav guanine nucleotide exchange factor 1 [Source:HGNC Symbol;Acc:HGNC:12657] | 601 | 0.622 | -0.0058 | No |
| 3 | PLCG2 | phospholipase C gamma 2 [Source:HGNC Symbol;Acc:HGNC:9066] | 725 | 0.588 | 0.0035 | No |
| 4 | ITGB2 | integrin subunit beta 2 [Source:HGNC Symbol;Acc:HGNC:6155] | 827 | 0.562 | 0.0137 | No |
| 5 | SIPA1 | signal-induced proliferation-associated 1 [Source:HGNC Symbol;Acc:HGNC:10885] | 1488 | 0.431 | -0.0241 | No |
| 6 | CLDN4 | claudin 4 [Source:HGNC Symbol;Acc:HGNC:2046] | 1716 | 0.395 | -0.0291 | No |
| 7 | ITGA4 | integrin subunit alpha 4 [Source:HGNC Symbol;Acc:HGNC:6140] | 1854 | 0.379 | -0.0276 | No |
| 8 | CTNNA3 | catenin alpha 3 [Source:HGNC Symbol;Acc:HGNC:2511] | 2030 | 0.358 | -0.0298 | No |
| 9 | PIK3CB | "phosphatidylinositol-4,5-bisphosphate 3-kinase catalytic subunit beta [Source:HGNC Symbol;Acc:HGNC:8976]" | 2186 | 0.340 | -0.0310 | No |
| 10 | NCF1 | neutrophil cytosolic factor 1 [Source:HGNC Symbol;Acc:HGNC:7660] | 2195 | 0.339 | -0.0207 | No |
| 11 | MAPK14 | mitogen-activated protein kinase 14 [Source:HGNC Symbol;Acc:HGNC:6876] | 2352 | 0.320 | -0.0226 | No |
| 12 | MYL2 | myosin light chain 2 [Source:HGNC Symbol;Acc:HGNC:7583] | 2376 | 0.317 | -0.0143 | No |
| 13 | ACTN2 | actinin alpha 2 [Source:HGNC Symbol;Acc:HGNC:164] | 2532 | 0.299 | -0.0168 | No |
| 14 | MAPK13 | mitogen-activated protein kinase 13 [Source:HGNC Symbol;Acc:HGNC:6875] | 2544 | 0.298 | -0.0080 | No |
| 15 | RHOH | ras homolog family member H [Source:HGNC Symbol;Acc:HGNC:686] | 2680 | 0.282 | -0.0095 | No |
| 16 | MYL5 | myosin light chain 5 [Source:HGNC Symbol;Acc:HGNC:7586] | 2705 | 0.279 | -0.0024 | No |
| 17 | MYLPF | "myosin light chain, phosphorylatable, fast skeletal muscle [Source:HGNC Symbol;Acc:HGNC:29824]" | 2816 | 0.268 | -0.0024 | No |
| 18 | TXK | TXK tyrosine kinase [Source:HGNC Symbol;Acc:HGNC:12434] | 2958 | 0.254 | -0.0053 | No |
| 19 | NCF4 | neutrophil cytosolic factor 4 [Source:HGNC Symbol;Acc:HGNC:7662] | 3036 | 0.246 | -0.0034 | No |
| 20 | MYL12A | myosin light chain 12A [Source:HGNC Symbol;Acc:HGNC:16701] | 3039 | 0.246 | 0.0044 | No |
| 21 | CLDN1 | claudin 1 [Source:HGNC Symbol;Acc:HGNC:2032] | 3089 | 0.241 | 0.0083 | No |
| 22 | GNAI3 | G protein subunit alpha i3 [Source:HGNC Symbol;Acc:HGNC:4387] | 3694 | 0.186 | -0.0330 | No |
| 23 | PRKCA | protein kinase C alpha [Source:HGNC Symbol;Acc:HGNC:9393] | 3728 | 0.183 | -0.0297 | No |
| 24 | EZR | ezrin [Source:HGNC Symbol;Acc:HGNC:12691] | 3817 | 0.174 | -0.0309 | No |
| 25 | CTNNA1 | catenin alpha 1 [Source:HGNC Symbol;Acc:HGNC:2509] | 4003 | 0.156 | -0.0404 | No |
| 26 | VAV3 | vav guanine nucleotide exchange factor 3 [Source:HGNC Symbol;Acc:HGNC:12659] | 4082 | 0.148 | -0.0417 | No |
| 27 | MYL10 | myosin light chain 10 [Source:HGNC Symbol;Acc:HGNC:29825] | 4125 | 0.144 | -0.0404 | No |
| 28 | CTNND1 | catenin delta 1 [Source:HGNC Symbol;Acc:HGNC:2515] | 4243 | 0.133 | -0.0453 | No |
| 29 | NOX1 | NADPH oxidase 1 [Source:HGNC Symbol;Acc:HGNC:7889] | 4438 | 0.118 | -0.0566 | No |
| 30 | MYL9 | myosin light chain 9 [Source:HGNC Symbol;Acc:HGNC:15754] | 4939 | 0.077 | -0.0932 | No |
| 31 | THY1 | Thy-1 cell surface antigen [Source:HGNC Symbol;Acc:HGNC:11801] | 4985 | 0.074 | -0.0944 | No |
| 32 | VASP | vasodilator stimulated phosphoprotein [Source:HGNC Symbol;Acc:HGNC:12652] | 5143 | 0.060 | -0.1047 | No |
| 33 | VCAM1 | vascular cell adhesion molecule 1 [Source:HGNC Symbol;Acc:HGNC:12663] | 5283 | 0.049 | -0.1140 | No |
| 34 | CLDN10 | claudin 10 [Source:HGNC Symbol;Acc:HGNC:2033] | 5479 | 0.034 | -0.1281 | No |
| 35 | JAM2 | junctional adhesion molecule 2 [Source:HGNC Symbol;Acc:HGNC:14686] | 5602 | 0.024 | -0.1369 | No |
| 36 | ITGAM | integrin subunit alpha M [Source:HGNC Symbol;Acc:HGNC:6149] | 5820 | 0.007 | -0.1536 | No |
| 37 | CLDN17 | claudin 17 [Source:HGNC Symbol;Acc:HGNC:2038] | 5931 | 0.000 | -0.1622 | No |
| 38 | ITK | IL2 inducible T cell kinase [Source:HGNC Symbol;Acc:HGNC:6171] | 6008 | -0.005 | -0.1680 | No |
| 39 | RAC2 | Rac family small GTPase 2 [Source:HGNC Symbol;Acc:HGNC:9802] | 6098 | -0.013 | -0.1746 | No |
| 40 | CXCR4 | C-X-C motif chemokine receptor 4 [Source:HGNC Symbol;Acc:HGNC:2561] | 6382 | -0.034 | -0.1956 | No |
| 41 | PRKCB | protein kinase C beta [Source:HGNC Symbol;Acc:HGNC:9395] | 6453 | -0.039 | -0.1998 | No |
| 42 | RAP1A | "RAP1A, member of RAS oncogene family [Source:HGNC Symbol;Acc:HGNC:9855]" | 6580 | -0.048 | -0.2081 | No |
| 43 | CYBB | cytochrome b-245 beta chain [Source:HGNC Symbol;Acc:HGNC:2578] | 6610 | -0.050 | -0.2088 | No |
| 44 | NCF2 | neutrophil cytosolic factor 2 [Source:HGNC Symbol;Acc:HGNC:7661] | 6871 | -0.069 | -0.2269 | No |
| 45 | MSN | moesin [Source:HGNC Symbol;Acc:HGNC:7373] | 6919 | -0.074 | -0.2282 | No |
| 46 | RAP1B | "RAP1B, member of RAS oncogene family [Source:HGNC Symbol;Acc:HGNC:9857]" | 6980 | -0.078 | -0.2304 | No |
| 47 | MMP2 | matrix metallopeptidase 2 [Source:HGNC Symbol;Acc:HGNC:7166] | 7037 | -0.082 | -0.2321 | No |
| 48 | AFDN | "afadin, adherens junction formation factor [Source:HGNC Symbol;Acc:HGNC:7137]" | 7077 | -0.086 | -0.2324 | No |
| 49 | MYL7 | myosin light chain 7 [Source:HGNC Symbol;Acc:HGNC:21719] | 7137 | -0.091 | -0.2341 | No |
| 50 | RAC1 | Rac family small GTPase 1 [Source:HGNC Symbol;Acc:HGNC:9801] | 7192 | -0.096 | -0.2352 | No |
| 51 | PTK2 | protein tyrosine kinase 2 [Source:HGNC Symbol;Acc:HGNC:9611] | 7315 | -0.103 | -0.2414 | No |
| 52 | ARHGAP5 | Rho GTPase activating protein 5 [Source:HGNC Symbol;Acc:HGNC:675] | 7357 | -0.106 | -0.2412 | No |
| 53 | PIK3CD | "phosphatidylinositol-4,5-bisphosphate 3-kinase catalytic subunit delta [Source:HGNC Symbol;Acc:HGNC:8977]" | 7405 | -0.111 | -0.2413 | No |
| 54 | CLDN8 | claudin 8 [Source:HGNC Symbol;Acc:HGNC:2050] | 7517 | -0.120 | -0.2462 | No |
| 55 | ACTN3 | actinin alpha 3 [Source:HGNC Symbol;Acc:HGNC:165] | 7557 | -0.124 | -0.2452 | No |
| 56 | VCL | vinculin [Source:HGNC Symbol;Acc:HGNC:12665] | 7687 | -0.133 | -0.2510 | No |
| 57 | PIK3CA | "phosphatidylinositol-4,5-bisphosphate 3-kinase catalytic subunit alpha [Source:HGNC Symbol;Acc:HGNC:8975]" | 7733 | -0.136 | -0.2502 | No |
| 58 | CLDN6 | claudin 6 [Source:HGNC Symbol;Acc:HGNC:2048] | 7883 | -0.147 | -0.2571 | No |
| 59 | PIK3CG | "phosphatidylinositol-4,5-bisphosphate 3-kinase catalytic subunit gamma [Source:HGNC Symbol;Acc:HGNC:8978]" | 7953 | -0.152 | -0.2576 | No |
| 60 | GNAI1 | G protein subunit alpha i1 [Source:HGNC Symbol;Acc:HGNC:4384] | 8110 | -0.164 | -0.2646 | No |
| 61 | MAPK12 | mitogen-activated protein kinase 12 [Source:HGNC Symbol;Acc:HGNC:6874] | 8161 | -0.168 | -0.2631 | No |
| 62 | ACTN1 | actinin alpha 1 [Source:HGNC Symbol;Acc:HGNC:163] | 8385 | -0.184 | -0.2746 | No |
| 63 | PRKCG | protein kinase C gamma [Source:HGNC Symbol;Acc:HGNC:9402] | 8582 | -0.200 | -0.2835 | Yes |
| 64 | RHOA | ras homolog family member A [Source:HGNC Symbol;Acc:HGNC:667] | 8601 | -0.201 | -0.2784 | Yes |
| 65 | GNAI2 | G protein subunit alpha i2 [Source:HGNC Symbol;Acc:HGNC:4385] | 8686 | -0.208 | -0.2783 | Yes |
| 66 | MMP9 | matrix metallopeptidase 9 [Source:HGNC Symbol;Acc:HGNC:7176] | 8698 | -0.209 | -0.2724 | Yes |
| 67 | PIK3R3 | phosphoinositide-3-kinase regulatory subunit 3 [Source:HGNC Symbol;Acc:HGNC:8981] | 8801 | -0.217 | -0.2735 | Yes |
| 68 | PIK3R5 | phosphoinositide-3-kinase regulatory subunit 5 [Source:HGNC Symbol;Acc:HGNC:30035] | 8804 | -0.217 | -0.2666 | Yes |
| 69 | CLDN11 | claudin 11 [Source:HGNC Symbol;Acc:HGNC:8514] | 8817 | -0.219 | -0.2606 | Yes |
| 70 | ARHGAP35 | Rho GTPase activating protein 35 [Source:HGNC Symbol;Acc:HGNC:4591] | 8835 | -0.220 | -0.2548 | Yes |
| 71 | CLDN14 | claudin 14 [Source:HGNC Symbol;Acc:HGNC:2035] | 9033 | -0.234 | -0.2627 | Yes |
| 72 | CLDN9 | claudin 9 [Source:HGNC Symbol;Acc:HGNC:2051] | 9203 | -0.248 | -0.2679 | Yes |
| 73 | NOX3 | NADPH oxidase 3 [Source:HGNC Symbol;Acc:HGNC:7890] | 9332 | -0.258 | -0.2696 | Yes |
| 74 | JAM3 | junctional adhesion molecule 3 [Source:HGNC Symbol;Acc:HGNC:15532] | 9376 | -0.262 | -0.2646 | Yes |
| 75 | ITGAL | integrin subunit alpha L [Source:HGNC Symbol;Acc:HGNC:6148] | 9526 | -0.276 | -0.2673 | Yes |
| 76 | ICAM1 | intercellular adhesion molecule 1 [Source:HGNC Symbol;Acc:HGNC:5344] | 9740 | -0.296 | -0.2745 | Yes |
| 77 | F11R | F11 receptor [Source:HGNC Symbol;Acc:HGNC:14685] | 9765 | -0.297 | -0.2668 | Yes |
| 78 | CLDN5 | claudin 5 [Source:HGNC Symbol;Acc:HGNC:2047] | 9816 | -0.302 | -0.2610 | Yes |
| 79 | PXN | paxillin [Source:HGNC Symbol;Acc:HGNC:9718] | 9845 | -0.305 | -0.2534 | Yes |
| 80 | CXCL12 | C-X-C motif chemokine ligand 12 [Source:HGNC Symbol;Acc:HGNC:10672] | 9901 | -0.312 | -0.2477 | Yes |
| 81 | CDH5 | cadherin 5 [Source:HGNC Symbol;Acc:HGNC:1764] | 9946 | -0.316 | -0.2410 | Yes |
| 82 | CD99 | CD99 molecule (Xg blood group) [Source:HGNC Symbol;Acc:HGNC:7082] | 10229 | -0.344 | -0.2519 | Yes |
| 83 | PECAM1 | platelet and endothelial cell adhesion molecule 1 [Source:HGNC Symbol;Acc:HGNC:8823] | 10304 | -0.351 | -0.2464 | Yes |
| 84 | ACTN4 | actinin alpha 4 [Source:HGNC Symbol;Acc:HGNC:166] | 10472 | -0.370 | -0.2476 | Yes |
| 85 | MYL12B | myosin light chain 12B [Source:HGNC Symbol;Acc:HGNC:29827] | 10505 | -0.374 | -0.2381 | Yes |
| 86 | ROCK2 | Rho associated coiled-coil containing protein kinase 2 [Source:HGNC Symbol;Acc:HGNC:10252] | 10648 | -0.388 | -0.2367 | Yes |
| 87 | PLCG1 | phospholipase C gamma 1 [Source:HGNC Symbol;Acc:HGNC:9065] | 10722 | -0.395 | -0.2297 | Yes |
| 88 | PIK3R2 | phosphoinositide-3-kinase regulatory subunit 2 [Source:HGNC Symbol;Acc:HGNC:8980] | 10956 | -0.425 | -0.2343 | Yes |
| 89 | PIK3R1 | phosphoinositide-3-kinase regulatory subunit 1 [Source:HGNC Symbol;Acc:HGNC:8979] | 11099 | -0.443 | -0.2311 | Yes |
| 90 | CLDN16 | claudin 16 [Source:HGNC Symbol;Acc:HGNC:2037] | 11104 | -0.444 | -0.2172 | Yes |
| 91 | CLDN7 | claudin 7 [Source:HGNC Symbol;Acc:HGNC:2049] | 11178 | -0.454 | -0.2083 | Yes |
| 92 | PTPN11 | protein tyrosine phosphatase non-receptor type 11 [Source:HGNC Symbol;Acc:HGNC:9644] | 11260 | -0.465 | -0.1997 | Yes |
| 93 | CTNNA2 | catenin alpha 2 [Source:HGNC Symbol;Acc:HGNC:2510] | 11342 | -0.477 | -0.1906 | Yes |
| 94 | RAPGEF3 | Rap guanine nucleotide exchange factor 3 [Source:HGNC Symbol;Acc:HGNC:16629] | 11380 | -0.483 | -0.1780 | Yes |
| 95 | VAV2 | vav guanine nucleotide exchange factor 2 [Source:HGNC Symbol;Acc:HGNC:12658] | 11413 | -0.489 | -0.1648 | Yes |
| 96 | MAPK11 | mitogen-activated protein kinase 11 [Source:HGNC Symbol;Acc:HGNC:6873] | 11501 | -0.501 | -0.1555 | Yes |
| 97 | PTK2B | protein tyrosine kinase 2 beta [Source:HGNC Symbol;Acc:HGNC:9612] | 11618 | -0.520 | -0.1479 | Yes |
| 98 | CLDN15 | claudin 15 [Source:HGNC Symbol;Acc:HGNC:2036] | 11849 | -0.559 | -0.1479 | Yes |
| 99 | RAPGEF4 | Rap guanine nucleotide exchange factor 4 [Source:HGNC Symbol;Acc:HGNC:16626] | 11963 | -0.586 | -0.1379 | Yes |
| 100 | ROCK1 | Rho associated coiled-coil containing protein kinase 1 [Source:HGNC Symbol;Acc:HGNC:10251] | 12046 | -0.605 | -0.1249 | Yes |
| 101 | CLDN18 | claudin 18 [Source:HGNC Symbol;Acc:HGNC:2039] | 12254 | -0.662 | -0.1198 | Yes |
| 102 | ITGB1 | integrin subunit beta 1 [Source:HGNC Symbol;Acc:HGNC:6153] | 12566 | -0.780 | -0.1190 | Yes |
| 103 | ACTG1 | actin gamma 1 [Source:HGNC Symbol;Acc:HGNC:144] | 12613 | -0.806 | -0.0967 | Yes |
| 104 | CLDN3 | claudin 3 [Source:HGNC Symbol;Acc:HGNC:2045] | 12615 | -0.807 | -0.0709 | Yes |
| 105 | CDC42 | cell division cycle 42 [Source:HGNC Symbol;Acc:HGNC:1736] | 12681 | -0.852 | -0.0486 | Yes |
| 106 | ACTB | actin beta [Source:HGNC Symbol;Acc:HGNC:132] | 12757 | -0.920 | -0.0249 | Yes |
| 107 | CTNNB1 | catenin beta 1 [Source:HGNC Symbol;Acc:HGNC:2514] | 12866 | -1.119 | 0.0027 | Yes |
Table: GSEA details [plain text format]

  

Fig 2: KEGG\_LEUKOCYTE\_TRANSENDOTHELIAL\_MIGRATION      
 Blue-Pink O' Gram in the Space of the Analyzed GeneSet

  

Fig 3: KEGG\_LEUKOCYTE\_TRANSENDOTHELIAL\_MIGRATION: Random ES distribution      
 Gene set null distribution of ES for **KEGG\_LEUKOCYTE\_TRANSENDOTHELIAL\_MIGRATION**
